# Pre-diagnostic vitamin D deficiency and subsequent thyroid cancer risk: a propensity-matched cohort study

**DOI:** 10.3389/fnut.2026.1817123

**Published:** 2026-04-21

**Authors:** Kuo-Chuan Hung, Chia-Li Kao, Ming Yew, Yi-Chen Lai, I-Wen Chen

**Affiliations:** 1 Department of Anesthesiology, Chi Mei Medical Center, Tainan City, Taiwan; 2 School of Medicine, College of Medicine, National Sun Yat-sen University, Kaohsiung, Taiwan; 3 Department of Anesthesiology, E-Da Hospital, I-Shou University, Kaohsiung City, Taiwan; 4 Department of Anesthesiology, Chi Mei Medical Center, Liouying, Tainan City, Taiwan

**Keywords:** 25-hydroxyvitamin D, nutritional epidemiology, thyroid cancer, thyroid neoplasm, vitamin D deficiency

## Abstract

**Background:**

Vitamin D deficiency (VDD) has been implicated as a potential risk factor for thyroid cancer; however, existing evidence is derived primarily from case-control or cross-sectional studies. This study aimed to examine the association between pre-diagnostic VDD and the risk of incident thyroid cancer over a 10-year follow-up period.

**Methods:**

This retrospective propensity score–matched cohort study used the TriNetX Global Collaborative Network. Adults aged ≥ 18 y with serum 25-hydroxyvitamin D [25(OH)D] measurements between 2010 and 2023 were classified as having VDD (<20 ng/mL) or vitamin D sufficiency (≥30 ng/mL). One-to-one propensity score matching was performed on demographics, comorbidities, thyroid-related disorders, laboratory values, and vitamin D supplementation. The primary outcome was incident thyroid cancer ascertained ≥ 180 day after the index date (landmark approach) over 10 y of follow-up. Positive (osteoporotic fracture) and negative (appendicitis, goiter) control outcomes and thyroid procedure utilization were evaluated to assess detection bias.

**Results:**

After matching, 571,669 pairs were analyzed over a median follow-up of 6.8 y. VDD was associated with an increased risk of incident thyroid cancer (HR: 1.41; 95% CI: 1.32, 1.50), and this risk was attenuated among patients with vitamin D insufficiency (25[OH]D 20–30 ng/mL; HR: 1.27; 95% CI: 1.20, 1.34), demonstrating a dose-dependent relationship. Thyroid biopsy and ultrasonography utilization were lower in the VDD cohort (HR: 0.95), arguing against detection bias. The positive control outcome (osteoporotic fracture; HR: 1.53) confirmed the validity of the exposure classification, whereas the negative control (appendicitis; HR: 1.02) showed no association. Findings were robust across all sensitivity and subgroup analyses.

**Conclusion:**

Pre-diagnostic VDD was associated with increased thyroid cancer risk in a dose-dependent manner. These findings support further investigation of vitamin D status as a potentially modifiable risk factor for thyroid cancer.

## 1 Introduction

Thyroid cancer is the most prevalent endocrine malignancy worldwide, with its incidence rising substantially over the past three decades (1–3). Although improved diagnostic surveillance partially accounts for this trend (2), genuine increases in disease burden have also been documented, underscoring the need to identify modifiable risk factors. Unlike many solid tumors, established modifiable risk factors for thyroid cancer remain limited, with radiation exposure being the most well-recognized contributor (4, 5). Vitamin D, a fat-soluble secosteroid hormone, has attracted considerable attention for its pleiotropic extraskeletal effects beyond classical calcium-phosphorus homeostasis (6, 7). By binding to the vitamin D receptor (VDR) expressed in thyroid follicular cells, the active metabolite 1,25-dihydroxyvitamin D exerts anti-proliferative, pro-differentiative, and pro-apoptotic effects, providing a biologically plausible basis for a protective role against thyroid carcinogenesis (8–11). However, epidemiological evidence examining this association has yielded inconsistent results. Several case-control studies and three meta-analyses have reported that lower serum 25-hydroxyvitamin D [25(OH)D] levels are associated with increased thyroid cancer risk, with pooled odds ratios ranging from 1.30 to 1.49 (12–15). Conversely, other investigations have found no significant association (16–18).

Several methodological limitations pervade existing evidence. First, most studies employed case-control or cross-sectional designs (12–15), in which vitamin D was measured at or after thyroid cancer diagnosis, precluding the establishment of temporal precedence and introducing reverse causation concerns. Second, the sample sizes were generally modest, limiting the statistical power for subgroup and dose-response analyses. Third, no prior study has systematically evaluated whether the observed association may be attributable to detection bias, whereby individuals with vitamin D deficiency (VDD) undergo more frequent thyroid surveillance. Fourth, reliance on single-point vitamin D measurements without assessment of exposure persistence raises uncertainty regarding chronic versus transient deficiencies.

To address these gaps, we conducted a large-scale propensity score-matched cohort study involving over one million patients with vitamin D status documented before outcome ascertainment. We incorporated positive and negative control outcomes, assessed detection bias through thyroid ultrasonography and biopsy utilization, evaluated dose-response relationships, and examined the persistence of VDD to strengthen causal inference within an observational framework.

## 2 Materials and methods

### 2.1 Study design and data source

This retrospective cohort analysis was performed using the TriNetX Global Collaborative Network, a federated research database that compiles de-identified electronic health records from 169 healthcare organizations spanning multiple countries. The database includes longitudinal patient-level information, such as demographic characteristics, diagnostic codes, procedural records, medication exposures, and laboratory measurements derived from both academic and community healthcare settings. TriNetX has been extensively utilized in observational research across various medical specialties and is recognized as a reliable source for large-scale real-world data analyses (19–22). The study protocol was reviewed and approved by the Institutional Review Board of Chi Mei Medical Center, with a waiver of informed consent owing to the use of anonymized, retrospective data.

### 2.2 Participant selection

Adult patients aged ≥ 18 years with at least one recorded serum 25-hydroxyvitamin D [25(OH)D] measurement between January 1, 2010, and December 31, 2023, were eligible for inclusion. Patients were included if any 25(OH)D measurement during the study period met the predefined exposure criteria. The index date was defined as the date of the first qualifying 25(OH)D measurement that satisfied cohort-specific criteria. Patients were categorized according to their qualifying 25(OH)D level: VDD was defined as < 20 ng/mL and control group as ≥ 30 ng/mL. Eligibility additionally required at least one thyroid-related healthcare utilization event (ultrasonography, fine-needle aspiration biopsy, or TSH testing) between 6 months and 10 years after the index date, thereby ensuring comparable surveillance opportunity across cohorts. To ensure exposure separation, individuals in the VDD group were excluded if they had any prior 25(OH)D level ≥ 30 ng/mL within the preceding 3 years, and those in the control group were excluded if they had any prior 25(OH)D level < 20 ng/mL during the same look-back period. Laboratory values in TriNetX are harmonized into standardized units across sites. Serum 25-hydroxyvitamin D levels were analyzed in ng/mL using uniform thresholds applied consistently across institutions, although inter-laboratory assay variability cannot be completely excluded.

### 2.3 Exclusion criteria

To establish an incident thyroid cancer cohort and minimize reverse causation and baseline confounding, several exclusions were applied. Patients with a diagnosis of malignant neoplasm of the thyroid gland (ICD-10-CM C73) prior to or within 6 months after the index date were excluded. Individuals who died within 6 months of the index date were excluded to ensure adequate follow-up (landmark analysis). Patients with chronic kidney disease stage 4–5, ESRD, or dialysis dependence prior to the index were excluded due to altered vitamin D metabolism. Additionally, patients with pregnancy, acute kidney injury, sepsis, severe sepsis, or receipt of critical care services within 1 month before the index date were excluded, as acute or critical illness may transiently alter serum 25(OH)D levels and may not reflect long-term vitamin D status. Patients with osteoporosis with current pathological fracture were also excluded at baseline because this outcome was prespecified as a positive control and therefore required exclusion to ensure incident case ascertainment.

### 2.4 Propensity score matching

Confounding was addressed through 1:1 propensity score matching implemented with a greedy nearest-neighbor approach without replacement. Propensity scores were derived from baseline variables assessed 3 years prior to the index date, including demographic factors (age, sex, race), body mass index, smoking status, comorbidities, thyroid-related disorders (hypothyroidism, hyperthyroidism, thyroiditis, goiter, and hyperparathyroidism), and family history of malignancy. Chronic liver disease was included as a baseline comorbidity in the propensity score matching model rather than used as an exclusion criterion. Baseline vitamin D supplementation was also incorporated. Laboratory parameters, including thyroid-stimulating hormone levels, were included to further address potential residual confounding. Covariate balance after matching was evaluated using standardized mean differences, with values < 0.1 considered indicative of adequate balance. Supplemental Table 1 show cohort construction, outcome definitions, and variables used for propensity score matching.

**TABLE 1:** Baseline characteristics of patients with vitamin D deficiency and vitamin D sufficiency before and after propensity score matching.

Variables	Before matching			After matching		
	VDD group (*n* = 664,823)	Control group (*n* = 1,376,733)	SMD†	VDD group (*n* = 571,669)	Control group (*n* = 571,669)	SMD†
Patient characteristics						
Age at index (years)	47.7 ± 18.1	56.5 ± 17.6	0.490	49.7 ± 17.8	50.2 ± 17.9	0.026
Female	474,623 (71.4)	1,029,993 (74.8)	0.077	409,466 (71.6)	405,887 (71.0)	0.014
BMI ≥ 30 kg/m^2^	252,875 (38.0)	391,526 (28.4)	0.205	202,213 (35.4)	207,249 (36.3)	0.018
White	411,601 (61.9)	1,140,315 (82.8)	0.481	405,020 (70.8)	403,067 (70.5)	0.008
Black or African American	153,707 (23.1)	107,671 (7.8)	0.433	85,100 (14.9)	90,159 (15.8)	0.025
Asian	27,346 (4.1)	42,745 (3.1)	0.054	24,483 (4.3)	23,038 (4.0)	0.013
Comorbidities						
Factors influencing health status and contact with health services	432,196 (65.0)	937,544 (68.1)	0.066	372,944 (65.2)	370,532 (64.8)	0.009
Essential (primary) hypertension	219,612 (33.0)	500,911 (36.4)	0.070	189,267 (33.1)	194,902 (34.1)	0.021
Dyslipidemia	195,560 (29.4)	534,229 (38.8)	0.199	178,410 (31.2)	183,063 (32.0)	0.018
Overweight and obesity	147,611 (22.2)	209,964 (15.3)	0.179	112,380 (19.7)	113,377 (19.8)	0.004
Neoplasms	119,045 (17.9)	327,680 (23.8)	0.145	108,234 (18.9)	107,515 (18.8)	0.003
Diabetes mellitus	102,581 (15.4)	191,411 (13.9)	0.043	85,268 (14.9)	86,732 (15.2)	0.007
Other hypothyroidism	78,139 (11.8)	237,459 (17.2)	0.157	73,727 (12.9)	72,898 (12.8)	0.004
Nicotine dependence	70,095 (10.5)	86,288 (6.3)	0.155	52,644 (9.2)	53,760 (9.4)	0.007
Other anemias	59,300 (8.9)	109,677 (8.0)	0.034	47,303 (8.3)	47,094 (8.2)	0.001
Obstructive sleep apnea	49,045 (7.4)	97,547 (7.1)	0.011	41,398 (7.2)	40,842 (7.1)	0.004
Ischemic heart diseases	45,196 (6.8)	111,462 (8.1)	0.049	40,337 (7.1)	40,532 (7.1)	0.001
Diseases of liver	35,071 (5.3)	66,311 (4.8)	0.021	29,984 (5.2)	29,548 (5.2)	0.003
Family history of primary malignant neoplasm	30,085 (4.5)	86,159 (6.3)	0.077	27,809 (4.9)	26,897 (4.7)	0.007
Nontoxic goiter	28,596 (4.3)	76,748 (5.6)	0.059	25,438 (4.5)	24,890 (4.4)	0.005
Chronic kidney disease (CKD)	26,651 (4.0)	68,811 (5.0)	0.048	23,626 (4.1)	23,710 (4.1)	0.001
Osteoporosis without current pathological fracture	22,042 (3.3)	119,592 (8.7)	0.228	21,741 (3.8)	21,912 (3.8)	0.002
Thyrotoxicosis	11,788 (1.8)	25,456 (1.8)	0.006	10,051 (1.8)	9,701 (1.7)	0.005
Encounter for screening for osteoporosis	9961 (1.5)	44,706 (3.2)	0.115	9744 (1.7)	9,800 (1.7)	0.001
Cerebral infarction	11,670 (1.8)	23,283 (1.7)	0.005	9,639 (1.7)	9,511 (1.7)	0.002
Thyroiditis	9,379 (1.4)	29,429 (2.1)	0.055	8,963 (1.6)	8,542 (1.5)	0.006
Iodine-deficiency related thyroid disorders and allied conditions	8039 (1.2)	16,093 (1.2)	0.004	6735 (1.2)	6,365 (1.1)	0.006
Malnutrition	7,871 (1.2)	12,806 (0.9)	0.025	6,400 (1.1)	6,101 (1.1)	0.005
Hyperparathyroidism and other disorders of parathyroid gland	5,200 (0.8)	16,200 (1.2)	0.040	4,825 (0.8)	4,682 (0.8)	0.003
Laboratory data						
Hemoglobin ≥ 12 g/dL	379,398 (57.1)	820,656 (59.6)	0.052	330,651 (57.8)	333,721 (58.4)	0.011
Albumin ≥ 3.5 g/dL	369,706 (55.6)	808,681 (58.7)	0.063	320,834 (56.1)	324,443 (56.8)	0.013
eGFR ≥ 60 mL/min/1.73 m^2^	360,132 (54.2)	763,524 (55.5)	0.026	308,996 (54.1)	312,985 (54.7)	0.014
TSH ≥ 4 m[IU]/L	54,890 (8.3)	121,618 (8.8)	0.021	49,338 (8.6)	48,341 (8.5)	0.006
Hemoglobin A1c ≥ 9%	31,200 (4.7)	32,075 (2.3)	0.129	21,779 (3.8)	22,051 (3.9)	0.002
Medication						
Vitamin D supplementation	68,826 (10.4)	234,818 (17.1)	0.196	64,863 (11.3)	68,017 (11.9)	0.017

Data are presented as mean ± standard deviation for continuous variables and n (%) for categorical variables. VDD, vitamin D deficiency; 25(OH)D, 25-hydroxyvitamin D; BMI, body mass index; CKD, chronic kidney disease; eGFR, estimated glomerular filtration rate; TSH, thyroid-stimulating hormone; HbA1c, hemoglobin A1c; SMD, standardized mean difference. † SMD values < 0.1 indicate adequate balance between groups after matching.

### 2.5 Outcome measures

The primary outcome was incident thyroid cancer (ICD-10-CM C73), while all-cause mortality was assessed as a secondary outcome to evaluate the overall long-term prognosis. To mitigate detection bias and reverse causation, outcome ascertainment commenced 180 days after the index date using a landmark approach, with follow-up extending up to 10 years. Patients were censored at the time of thyroid cancer diagnosis, death, last recorded healthcare encounter, or end of follow-up, whichever occurred first.

To evaluate the validity of our findings, osteoporotic fracture served as a positive control outcome, given its established association with VDD, whereas appendicitis and goiter served as negative control outcomes. Thyroid biopsy and ultrasonography utilization were compared between the groups to assess potential detection bias. Additionally, outcomes were evaluated at the 3-year follow-up to examine whether the association was driven by early detection; if the elevated risk was confined to the initial follow-up period and attenuated over time, this would suggest that increased medical surveillance rather than a biological effect may account for the observed association. Finally, to address the limitation that a single baseline measurement may not reflect long-term vitamin D status, we assessed the risk of subsequent VDD (25[OH]D < 20 ng/mL) during the 6-month to 10-year follow-up period, comparing the VDD and control cohorts.

### 2.6 Sensitivity and subgroup analyses

Three sensitivity analyses were performed to evaluate the robustness of the primary findings. In Model I, patients who died during the 10-year follow-up were excluded to determine whether competing risks from mortality influenced the observed association. Model II restricted the analysis to patients enrolled between 2015 and 2023 to reflect contemporary clinical practice and diagnostic patterns. Model III excluded patients with any prior cancer history to minimize confounding from pre-existing malignancies that may independently influence both vitamin D metabolism and thyroid cancer surveillance. Pre-specified subgroup analyses examined the potential effect modification by age (18–40 vs. > 41 years), sex, diabetes mellitus status, obesity, smoking history, and baseline thyroid conditions (goiter and thyroiditis).

### 2.7 Dose-dependent analysis

To explore a potential dose-dependent relationship between vitamin D status and thyroid cancer risk, a separate propensity score-matched analysis was conducted to compare patients with vitamin D insufficiency (25[OH]D 20–30 ng/mL) to those in control group. By evaluating whether a lesser degree of vitamin D inadequacy was associated with a correspondingly attenuated risk compared with the primary analysis of VDD ( < 20 ng/mL), this analysis aimed to assess whether a biological gradient exists between decreasing vitamin D levels and increasing thyroid cancer risk.

### 2.8 Statistical approach

Missing covariate data were addressed through available-case analysis. Cox proportional hazards models were used to derive hazard ratios (HRs) with 95% confidence intervals (CIs) for all time-to-event outcomes. Cumulative incidence was illustrated using Kaplan-Meier curves. The proportional hazard assumption was evaluated using Schoenfeld residual tests. No adjustment for multiple comparisons was applied, as the outcomes were hierarchically structured with a single pre-specified primary endpoint (thyroid cancer), while secondary and control outcomes served distinct confirmatory or validation roles rather than representing independent hypotheses of equal weight. All tests were two-sided, with statistical significance set at p < 0.05. Analyses were conducted using the built-in analytical tools of the TriNetX platform.

## 3 Results

### 3.1 Patient selection and baseline characteristics

**FIGURE 1:**
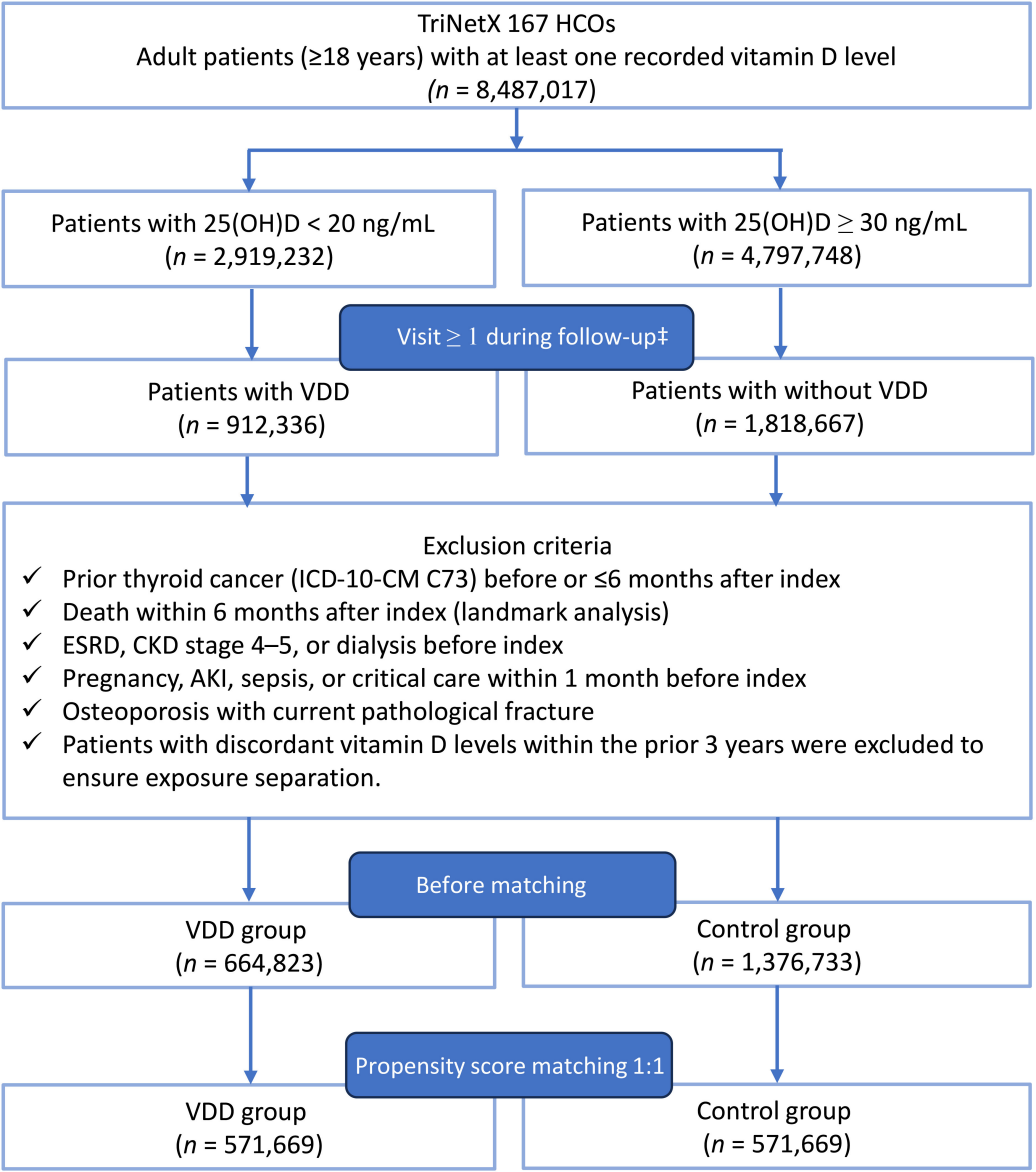
Patient selection flowchart. Adults with serum 25-hydroxyvitamin D measurements between 2010 and 2023 were categorized into the vitamin D deficiency (VDD) (<20 ng/mL) and vitamin D sufficiency (≥30 ng/mL) cohorts. VDD, vitamin D deficiency; 25(OH)D, 25-hydroxyvitamin D; HCOs, healthcare organizations; ESRD, end-stage renal disease; CKD, chronic kidney disease; AKI, acute kidney injury; PSM, propensity score matching; ^‡^Eligibility additionally required at least one thyroid-related healthcare utilization event (ultrasonography, fine-needle aspiration biopsy, or TSH testing) between 6 months and 10 years after the index date

The patient selection process is shown in Figure 1. From the TriNetX Global Collaborative Network, 664,823 patients met the criteria for VDD, and 1,376,733 met the criteria for vitamin D sufficiency. After 1:1 propensity score matching, 571,669 patients were included in each cohort. Before matching, the VDD cohort was younger (47.7 ± 18.1 vs. 56.5 ± 17.6 years), had a higher proportion of Black or African American individuals (23.1% vs. 7.8%), and a greater prevalence of obesity (22.2% vs. 15.3%). After matching, all standardized mean differences were below 0.1, indicating an adequate covariate balance across demographics, comorbidities, thyroid-related disorders, laboratory values, and vitamin D supplementation (Table 1).

### 3.2 Association between VDD and 10-year outcomes

**FIGURE 2:**
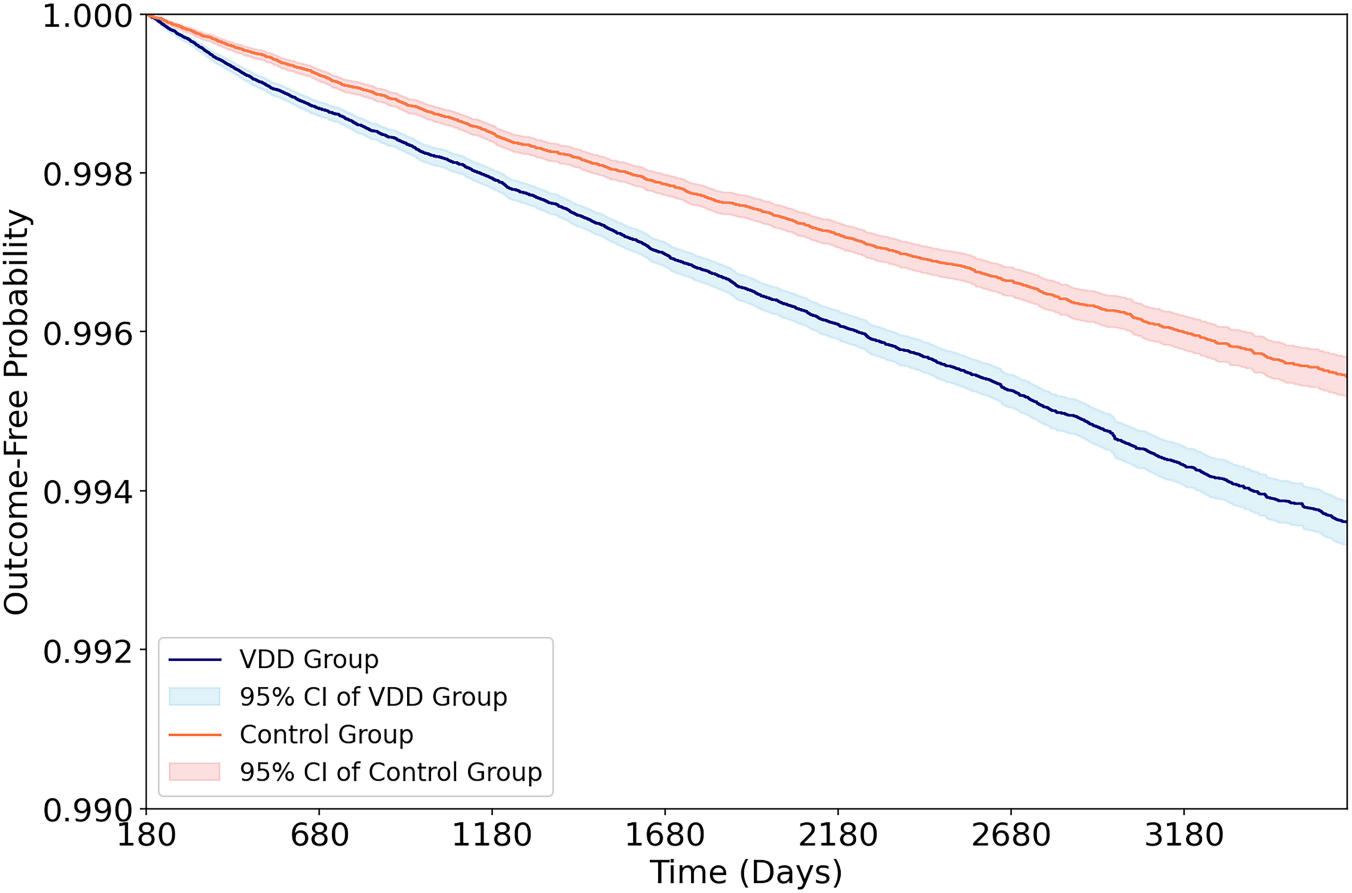
Kaplan–Meier cumulative incidence curves for thyroid cancer in the vitamin D deficiency (VDD) and patients without VDD (control cohorts) during the 10-year follow-up. The outcome assessment commenced 180 days after the index date (landmark approach). Shaded areas represent 95% confidence intervals. VDD, vitamin D deficiency; HR, hazard ratio; CI, confidence interval.

The median follow-up duration was 6.8 years (IQR 5.5 years) in the VDD cohort and 6.1 years (IQR 5.4 years) in the control cohort, corresponding to mean follow-up times of 6.6 ± 2.8 and 6.2 ± 2.8 years, respectively. Over a maximum follow-up of 10 years following the 6-month landmark period, VDD was associated with a significantly elevated risk of thyroid cancer compared with vitamin D sufficiency (0.43% vs. 0.29%; HR 1.41, 95% CI 1.32–1.50, *p* < 0.001) (Figure 2 and Table 2). VDD was also associated with increased all-cause mortality (HR 1.38, *p* < 0.001). The positive control outcome, osteoporotic fracture, demonstrated a robust association with VDD (HR 1.53, *p* < 0.001), confirming the biological plausibility of the exposure classification.

**TABLE 2:** Association between vitamin D deficiency and clinical outcomes during the 10-year follow-up in propensity score-matched cohorts.

Outcomes	VDD group^‡^ Events (%)	Control group^‡^ Events (%)	HR (95% CI)	*P*-value
Thyroid cancer	2,435 (0.43%)	1,637 (0.29%)	1.41 (1.32–1.50)	< 0.001
Mortality	40,295 (7.05%)	27,332 (4.78%)	1.38 (1.36–1.40)	< 0.001
Osteoporotic fracture (positive control)	6,202 (1.09%)	3,791 (0.66%)	1.53 (1.47–1.59)	< 0.001
Appendicitis (negative control)	4,511 (0.79%)	4,190 (0.73%)	1.02 (0.98–1.06)	0.780
Goiter (negative control)	64,261 (11.24%)	64,413 (11.27%)	0.96 (0.95–0.97)	< 0.001
Thyroid biopsy/US utilization	75,305 (13.17%)	76,011 (13.30%)	0.95 (0.94–0.96)	< 0.001

Values are presented as the number of events (percentage). ^‡^Each cohort included 571,669 patients after 1:1 propensity score matching. The outcome assessment commenced 180 days after the index date (landmark approach). VDD, vitamin D deficiency; HR, hazard ratio; CI, confidence interval; US, ultrasonography.

In contrast, negative control outcomes did not demonstrate clinically meaningful increases. Appendicitis was not significantly associated with VDD (HR 1.02, *p* = 0.780), while goiter showed a marginally lower risk in the VDD group (HR 0.96, *p* < 0.001). Importantly, thyroid biopsy and ultrasonography utilization were not higher in the VDD cohort (HR 0.95, *p* < 0.001), indicating that the elevated thyroid cancer risk was unlikely attributable to differential diagnostic surveillance.

To evaluate whether the observed association was driven by early detection bias, the outcomes were assessed at the 3-year follow-up (Table 3). VDD remained significantly associated with thyroid cancer at 3 years (HR 1.32, *p* < 0.001), and this point estimate was numerically lower than the 10-year estimate (HR 1.41), suggesting that the association was not concentrated in the early post-index period but rather persisted and strengthened over time.

**TABLE 3:** Association between vitamin D deficiency and clinical outcomes during 3-year follow-up in propensity score-matched cohorts.

Outcomes	VDD group^‡^ Events (%)	Control group^‡^ Events (%)	HR (95% CI)	*P*-value
Thyroid cancer	1,050 (0.18%)	793 (0.14%)	1.32 (1.20–1.45)	< 0.001
Mortality	13,417 (2.35%)	8,854 (1.55%)	1.51 (1.47–1.55)	< 0.001
Osteoporotic fracture (positive control)	2,048 (0.36%)	1,168 (0.20%)	1.75 (1.62–1.88)	< 0.001
Appendicitis (negative control)	1,897 (0.33%)	1,843 (0.32%)	1.03 (0.96–1.09)	0.444
Goiter (negative control)	39,320 (6.88%)	42,031 (7.35%)	0.93 (0.92–0.94)	< 0.001
Thyroid biopsy/US utilization	39,981 (6.99%)	43,541 (7.62%)	0.91 (0.90–0.92)	0.029

Values are presented as number of events (percentage). ^‡^Each cohort included 571,717 patients after 1:1 propensity score matching. Outcome assessment commenced 180 days after the index date (landmark approach). VDD, vitamin D deficiency; HR, hazard ratio; CI, confidence interval; US, ultrasonography.

### 3.3 Sensitivity and subgroup analyses

All three sensitivity analyses yielded results consistent with the primary analysis (Table 4). The association between VDD and thyroid cancer persisted when excluding patients who died during follow-up (Model I: HR 1.41, *p* < 0.001), when restricting to the 2015–2023 enrollment period (Model II: HR 1.38, *p* < 0.001), and when excluding patients with any prior cancer history (Model III: HR 1.30, *p* < 0.001).

**TABLE 4:** Sensitivity analyses for the association between vitamin D deficiency and clinical outcomes during 10-year follow-up.

Outcomes	Model I		Model II		Model III	
	HR (95% CI)	*p*-value	HR (95% CI)	*p*-value	HR (95% CI)	*p*-value
Thyroid cancer	1.41 (1.32, 1.51)	< 0.001	1.38 (1.29, 1.49)	<0.001	1.30 (1.21, 1.40)	<0.001
Mortality	NA	NA	1.40 (1.38, 1.43)	<0.001	1.35 (1.32, 1.37)	<0.001
Osteoporotic fracture	1.53 (1.46, 1.60)	<0.001	1.46 (1.40, 1.53)	<0.001	1.45 (1.38, 1.53)	<0.001
Appendicitis (negative control)	0.98 (0.94, 1.02)	0.621	1.04 (0.99, 1.09)	0.111	1.00 (0.95, 1.05)	0.908
Goiter (negative control)	0.97 (0.95, 0.98)	<0.001	0.96 (0.95, 0.98)	<0.001	0.97 (0.95, 0.98)	<0.001
Thyroid biopsy/US utilization	0.95 (0.94, 0.96)	<0.001	0.95 (0.94, 0.96)	<0.001	0.94 (0.93, 0.95)	<0.001

Model I excluded patients who died during the 10-year follow-up (*n* = 530,239 per group). Model II restricted the analysis to patients enrolled between 2015 and 2023 to reflect contemporary clinical practice (*n* = 497,396 per group). Model III excluded patients with any prior cancer history (*n* = 441,625 per group). The outcome assessment commenced 180 days after the index date (landmark approach). HR, hazard ratio; CI, confidence interval; US, ultrasonography; NA, not applicable.

Subgroup analyses demonstrated a consistent association between VDD and thyroid cancer across all prespecified strata (Table 5). The association was significant in both younger (18–40 years: HR 1.27, *p* = 0.005) and older (>41 years: HR 1.41, *p* < 0.001) patients in both sexes, regardless of obesity, smoking, or diabetes mellitus status. No significant interaction was observed for any subgroup variable (all *p* > 0.05). Notably, numerically higher point estimates were observed among patients with diabetes mellitus (HR, 1.58) and those with baseline thyroiditis (HR, 1.68), although the interaction tests did not reach statistical significance.

**TABLE 5:** Subgroup analyses for the association between vitamin D deficiency and thyroid cancer during the 10-year follow-up.

Subgroup	VDD group (n)	Control group (n)	HR (95% CI)	*P*-value	P for interaction
Overall age					
18–40 years	114,970	114,970	1.27 (1.08, 1.50)	0.005	Reference
> 1 years	502,744	502,744	1.41 (1.32, 1.50)	<0.001	0.230
Gender					
Male	163,483	163,483	1.38 (1.20, 1.59)	<0.001	Reference
Female	348,158	348,158	1.40 (1.30, 1.52)	<0.001	0.861
Diabetes mellitus (DM)					
With DM	110,330	110,330	1.58 (1.36, 1.83)	<0.001	Reference
Without DM	502,842	502,842	1.35 (1.26, 1.44)	<0.001	0.073
Obesity					
With obesity	293,454	293,454	1.33 (1.23, 1.45)	<0.001	Reference
Without obesity	288,488	288,488	1.42 (1.29, 1.55)	<0.001	0.300
Smoking (nicotine)					
Ever smoker	77,757	77,757	1.28 (1.06, 1.54)	0.009	Reference
Non-smoker	521,288	521,288	1.41 (1.32, 1.50)	<0.001	0.320
Baseline thyroid status					
Goiter	42,182	42,182	1.49 (1.30, 1.72)	<0.001	Reference
No goiter	530,830	530,830	1.41 (1.31, 1.51)	<0.001	0.500
Thyroiditis	29,040	29,040	1.68 (1.35, 2.09)	<0.001	Reference
No thyroiditis	563,114	563,114	1.36 (1.28, 1.45)	<0.001	0.099

VDD, vitamin D deficiency; HR, hazard ratio; CI, confidence interval; DM, diabetes mellitus.

### 3.4 Dose-response analysis

The dose-response analysis comparing vitamin D insufficiency (25[OH]D 20–30 ng/mL) with sufficiency (≥30 ng/mL) demonstrated a significant but attenuated association with thyroid cancer (HR 1.27, *p* < 0.001) (Table 6) compared with the primary analysis of VDD (<20 ng/mL; HR 1.41). This graded pattern, whereby more severe vitamin D inadequacy corresponded to a greater magnitude of risk, supports a dose-dependent relationship. The control outcomes in this analysis were consistent with the primary findings: osteoporotic fractures remained elevated (HR 1.37, *p* < 0.001), appendicitis showed no association (HR 1.01, *p* = 0.108), and goiter and thyroid biopsy/ultrasonography utilization were not increased.

**TABLE 6:** Association between vitamin D insufficiency and clinical outcomes during the 10-year follow-up in propensity score-matched cohorts.

Outcomes	VDI group^‡^ Events (%)	Control group^‡^ Events (%)	HR (95% CI)	*P*-value
Thyroid cancer	2,923 (0.40%)	2,207 (0.30%)	1.27 (1.20–1.34)	<0.001
Mortality	45,291 (6.20%)	36,518 (5.00%)	1.18 (1.16–1.19)	<0.001
Osteoporotic fracture (positive control)	8,019 (1.10%)	5,523 (0.76%)	1.37 (1.33–1.42)	<0.001
Appendicitis (negative control)	5,699 (0.78%)	5,391 (0.74%)	1.01 (0.97–1.05)	0.108
Goiter (negative control)	82,830 (11.33%)	81,149 (11.10%)	0.99 (0.98–1.00)	0.101
Thyroid biopsy/US utilization	96,886 (13.25%)	95,735 (13.10%)	0.98 (0.97–0.99)	<0.001

VDI: Vitamin D insufficiency was defined as 25(OH)D 20–30 ng/mL, and vitamin D sufficiency as 25(OH)D ≥ 30 ng/mL. Values are presented as the number of events (percentage). ^‡^Each cohort included 731,096 patients after 1:1 propensity score matching. The outcome assessment commenced 180 days after the index date (landmark approach). 25(OH)D, 25-hydroxyvitamin D; HR, hazard ratio; CI, confidence interval; US, ultrasonography.

### 3.5 Persistence of vitamin D deficiency

**FIGURE 3:**
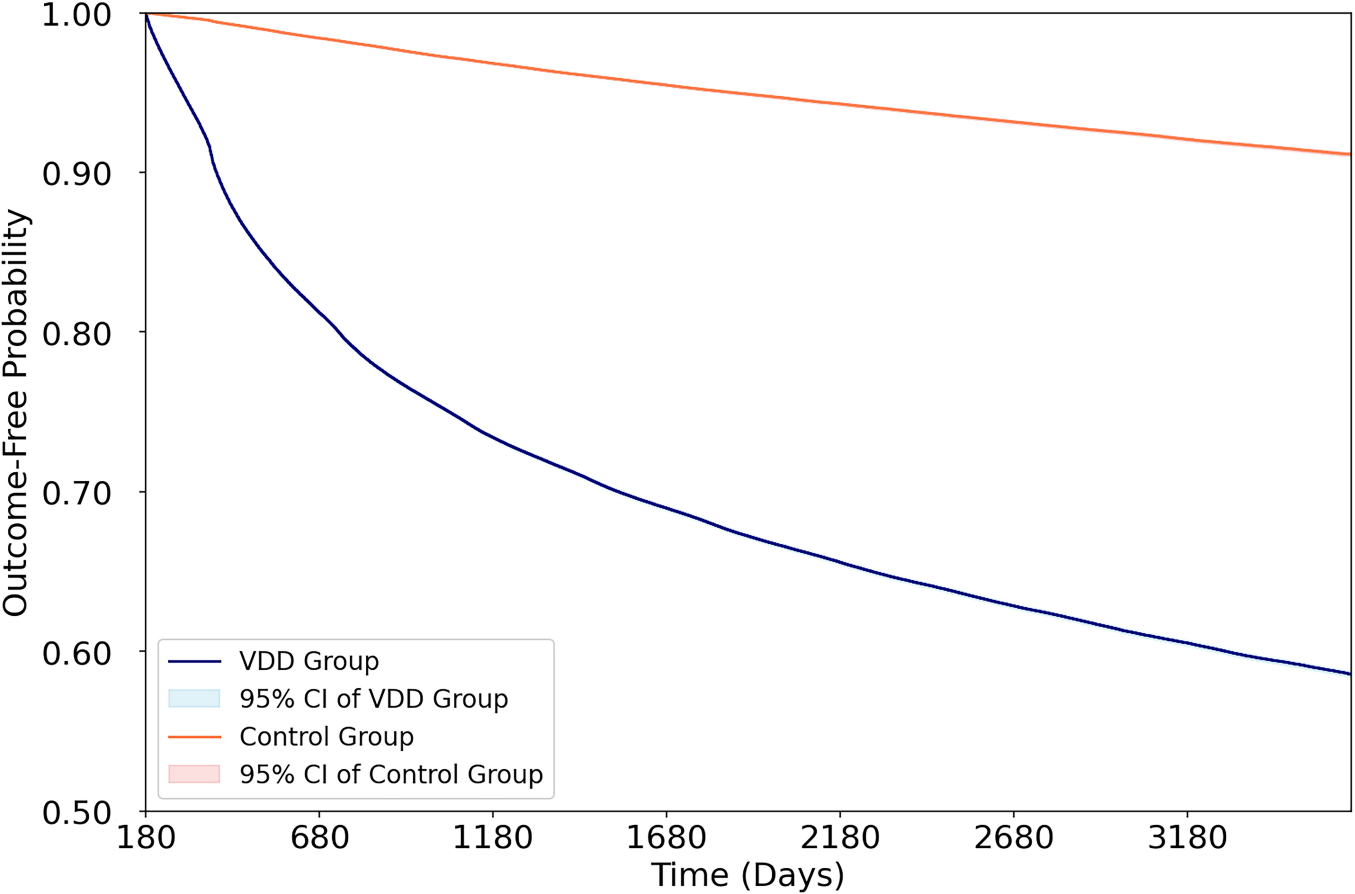
Kaplan–Meier cumulative incidence curves for subsequent vitamin D deficiency (25[OH]D < 20 ng/mL) during the 6-month to 10-year follow-up period. Patients with baseline vitamin D deficiency demonstrated a markedly higher risk of subsequent vitamin D deficiency compared with controls (HR 7.02, 95% CI 6.94–7.10, p < 0.001), supporting the persistence of the exposure over time. VDD, vitamin D deficiency; 25(OH)D, 25-hydroxyvitamin D; HR, hazard ratio; CI, confidence interval.

To address the concern that a single baseline 25(OH)D measurement may not represent long-term vitamin D status, the risk of subsequent VDD during the 6-month to 10-year follow-up was evaluated. Patients with baseline VDD had a markedly elevated risk of subsequent VDD compared with controls (34.3% vs. 5.8%, HR 7.02, 95% CI 6.94–7.10, *p* < 0.001) (Figure 3), indicating that baseline VDD strongly predicted persistent VDD throughout the follow-up period. This finding supports the validity of using a single baseline measurement as a surrogate for chronic vitamin D status in this population.

## 4 Discussion

In this multi-institutional propensity score-matched cohort study encompassing over 1.1 million adults, VDD was associated with a 41% higher risk of incident thyroid cancer over a decade of follow-up. This association exhibited a dose-dependent pattern, with vitamin D insufficiency conferring an attenuated but still significant elevation in risk. The findings remained robust across multiple sensitivity analyses, were consistent across demographic and clinical subgroups, and could not be explained by differential thyroid surveillance or detection biases. Furthermore, a single baseline measurement of VDD strongly predicted persistent deficiency throughout the follow-up period, supporting the validity of the exposure assessment. Collectively, these findings provide the most comprehensive observational evidence to date linking low vitamin D levels to the risk of thyroid cancer.

Evidence from meta-analyses suggested that VDD may be associated with thyroid cancer risk by approximately 33–49% (12–15), but the evidence was hampered by substantial heterogeneity and methodological inconsistencies across studies, including variable vitamin D thresholds, different assay methods, and inconsistent adjustment for confounders. Furthermore, the relationship between vitamin D status and thyroid cancer has been investigated primarily through case-control studies or cross-sectional designs with limited sample sizes (12–15). Critically, most studies included in previous meta-analyses (12–15) measured vitamin D at or after thyroid cancer diagnosis, making it impossible to disentangle whether low vitamin D preceded or resulted from malignancy. Additionally, no previous study has evaluated whether increased healthcare utilization among vitamin D-deficient individuals, rather than a true biological effect, could explain the observed association. Our study addresses these knowledge gaps by employing a longitudinal cohort design with pre-diagnostic vitamin D assessment, rigorous propensity score matching for over 25 covariates, including thyroid-related conditions, and systematic evaluation of detection bias through control outcomes and thyroid procedure utilization. Although causality cannot be established in an observational study, the novelty of the present study lies in its pre-diagnostic longitudinal design, large propensity-matched cohort, dose-response analysis, and formal evaluation of detection bias and control outcomes, which extend substantially beyond prior predominantly case-control or cross-sectional studies.

The observed 41% increased risk of thyroid cancer among individuals with VDD was identified in a large propensity score–matched cohort comprising 571,669 pairs, making this one of the largest longitudinal studies to date with pre-diagnostic vitamin D assessment. Several methodological considerations have enhanced the robustness of this finding. First, the 6-month landmark design reduced the likelihood of reverse causation by excluding thyroid cancers diagnosed shortly after vitamin D measurement. Second, the large sample size provided adequate statistical power for both the primary analysis and pre-specified subgroup analyses across age, sex, diabetes, obesity, smoking status, and baseline thyroid disorders, none of which showed significant effect modification. Third, our results remained consistent across multiple sensitivity analyses. The persistence of the association after excluding patients with pre-existing cancer further reduces the possibility that malignancy-related metabolic changes or surveillance differences substantially influence the primary estimate. The dose-response analysis further reinforced the biological plausibility of this association. Vitamin D insufficiency (25[OH]D 20–30 ng/mL) was associated with a 27% increased thyroid cancer risk compared with 41% for deficiency (<20 ng/mL), demonstrating a graded relationship between decreasing vitamin D levels and increasing cancer risk that fulfills the Bradford Hill criterion of the biological gradient.

The biological plausibility of this association is supported by several mechanisms. Through its active metabolite, 1,25-dihydroxyvitamin D, vitamin D binds the vitamin D receptor in thyroid follicular cells and exerts anti-proliferative, pro-differentiative, and pro-apoptotic effects that may inhibit thyroid carcinogenesis (8–10). Beyond these direct cellular effects, vitamin D also modulates innate and adaptive immunity, including T-helper cell differentiation, regulatory T-cell activity, and pro-inflammatory cytokine signaling (23–25). This is relevant because autoimmune thyroid diseases, particularly Hashimoto’s thyroiditis, have been linked to increased thyroid cancer risk (26, 27); thus, VDD may also promote carcinogenesis indirectly by facilitating chronic thyroid inflammation. Consistent with this possibility, our subgroup analysis showed a numerically higher point estimate among patients with baseline thyroiditis (HR 1.68), although the interaction was not statistically significant. It should also be noted that some studies have reported no significant association between vitamin D status and thyroid cancer (16–18). These discrepant findings may reflect differences in study design, sample size, assay methods, population characteristics, and the timing of vitamin D measurement relative to cancer diagnosis, particularly in studies with post-diagnostic assessment that are more vulnerable to reverse causation.

In the current study, an important methodological consideration was defining the index date as the first 25(OH)D measurement meeting the cohort-specific threshold, rather than the chronologically first available vitamin D value. Using the earliest measurement, irrespective of its level, could have classified patients based on borderline or transient values that may not reflect habitual vitamin D status. Additionally, early measurements may occur during acute illness, perioperative evaluation, or routine screening, potentially introducing exposure misclassification. By requiring a definitive threshold (<20 ng/mL for deficiency or ≥ 30 ng/mL for sufficiency) and excluding individuals with discordant values within the preceding 3 years, we aimed to enhance exposure specificity and improve separation between groups. This approach prioritizes biological relevance over a purely temporal definition while reducing potential misclassification.

The incorporation of positive and negative control outcomes provided additional validation of our findings. Osteoporotic fracture, a condition with well-established links to VDD, showed a significantly elevated risk (HR, 1.53), confirming that the exposure classification accurately captured clinically meaningful VDD. Conversely, appendicitis, a condition with no known biological relationship with vitamin D, showed no association, suggesting that the observed thyroid cancer risk was not driven by a generalized increase in healthcare encounters or diagnostic coding among vitamin D-deficient patients. Notably, the risk of goiter was not elevated in the VDD group (HR 0.96), and thyroid biopsy and ultrasonography utilization were lower in the VDD cohort (HR 0.95). These findings argue against the possibility that vitamin D-deficient individuals undergo more intensive thyroid surveillance, which could otherwise increase thyroid cancer detection. Furthermore, at the 3-year follow-up, the hazard ratio for thyroid cancer was 1.32, compared with 1.41 at 10 years. This gradual increase over time suggests that the association was not concentrated in the early post-index period, a pattern less consistent with immediate detection bias, and more compatible with a sustained or cumulative effect.

A common criticism of observational studies relying on single vitamin D measurements is that a single value may not accurately represent long-term vitamin D status. Our analysis directly addressed this concern by demonstrating that patients with baseline VDD had a seven-fold higher risk of subsequent VDD during follow-up (HR 7.02) than controls, with over one-third of VDD patients experiencing recurrent deficiency versus fewer than 6% of controls. This substantial disparity indicates that baseline VDD is not a transient phenomenon, but rather reflects a chronic state of inadequacy, likely driven by persistent behavioral, dietary, and environmental factors.

Several limitations should be acknowledged. First, the observational design precludes causal inference, and residual confounding by unmeasured factors such as ultraviolet exposure, dietary patterns, physical activity, and socioeconomic status cannot be excluded despite comprehensive propensity score matching. Second, the TriNetX database relies on diagnostic codes, which may introduce exposure or outcome misclassification; however, we employed established ICD-10-CM codes and laboratory-based vitamin D thresholds to minimize this concern. Third, the TriNetX database does not provide detailed oncological information such as tumor stage, histological subtype, or complete treatment details, including radioiodine therapy. Therefore, we were unable to evaluate disease stage, treatment modalities, or cancer-specific outcomes, and our analysis was limited to incident thyroid cancer diagnoses identified by ICD codes. Fourth, another limitation is that vitamin D status was primarily defined based on a single baseline 25(OH)D measurement. However, our analysis showed that patients with baseline VDD had a substantially higher risk of subsequent VDD during follow-up, suggesting that baseline measurements likely reflect persistent rather than transient deficiency in many patients. Nevertheless, serial vitamin D measurements were not available for all individuals, and future studies with repeated measurements are warranted. Fifth, the TriNetX platform does not support continuous variable modeling, precluding more granular dose-response analyses; future studies using individual-level data with serial 25(OH)D measurements should explore additional thresholds and nonlinear relationships to further characterize the shape of the dose-response curve. Finally, although we adjusted for vitamin D supplementation at baseline, we could not account for changes in supplementation during the follow-up period. In addition, subgroup analysis based on TSH levels were not performed because TSH laboratory data were available only for a subset of patients, which would substantially reduce statistical power and potentially introduce selection bias.

## 5 Conclusion

In this large propensity-score-matched cohort study, VDD was associated with a significantly increased risk of incident thyroid cancer over 10 years of follow-up. This association demonstrated a dose-response relationship, persisted across sensitivity and subgroup analyses, and was not attributable to detection bias. However, the observational nature of this study precludes causal conclusions. Future research should include prospective studies with repeated vitamin D measurements, mechanistic studies to clarify biological pathways, and randomized trials to determine whether correction of VDD can reduce thyroid cancer risk. Studies examining thyroid cancer subtypes and different populations are also warranted.

## Data Availability

The raw data supporting the conclusions of this article will be made available by the authors, without undue reservation.
