# Supplementary material for: Pre-diagnostic vitamin D deficiency and subsequent thyroid cancer risk: a propensity-matched cohort study
Source: Front Nutr. 2026 Apr 21;13:1817123. doi: 10.3389/fnut.2026.1817123 (PMC13138976; doi:10.3389/fnut.2026.1817123)
Supplement: Supplementary file 1 [file Table_1.docx]

**Supplemental Table 1.** Cohort Construction, Outcome Definitions, and Variables Used for Propensity Score Matching

| Category | Variable / Definition | Code / Specification |
| --- | --- | --- |
| Inclusion Criteria | Age ≥18 years at index | Demographics: Age |
|  | Vitamin D Deficiency cohort: first 25(OH)D <20 ng/mL (2010–2023) | TNX:9034; TNX:LG25965-1 <20 ng/mL |
|  | Control cohort: first 25(OH)D ≥30 ng/mL (2010–2023) | TNX:9034; TNX:LG25965-1 ≥30 ng/mL |
|  | Thyroid-related evaluation after index (6 months–10 years) | CPT 76536; CPT 10005; CPT 10021; CPT 84443 |
| Exclusion Criteria | Pre-existing thyroid cancer | ICD-10-CM C73 |
|  | Osteoporosis with pathological fracture | ICD-10-CM M80 |
|  | End-stage renal disease | ICD-10-CM N18.6 |
|  | CKD stage 4 or 5 | ICD-10-CM N18.4; N18.5 |
|  | Dialysis dependence | ICD-10-CM Z99.2 |
|  | Pregnancy | ICD-10-PCS 10; ICD-10-CM Z33.1; LOINC 82810-3 |
|  | Critical care services (≤1 month pre-index) | CPT 1013729 |
|  | Acute kidney injury | ICD-10-CM N17 |
|  | Severe sepsis | ICD-10-CM R65.2 |
|  | Sepsis | ICD-10-CM A41 |
|  | Early mortality (≤6 months) | Deceased; ICD-10-CM R99 |
|  | Prior high vitamin D (VDD cohort) | TNX:9034; TNX:LG25965-1 ≥30 ng/mL within 3 years |
|  | Prior low vitamin D (Control cohort) | TNX:9034; TNX:LG25965-1 <20 ng/mL within 3 years |
| Primary Outcome | Thyroid cancer | ICD-10-CM C73 |
| Secondary Outcomes | Mortality | Deceased; ICD-10-CM R99 |
|  | Fracture | ICD-10-CM M80 |
|  | Goiter | ICD-10-CM E04 |
|  | Appendicitis | ICD-10-CM K35–K38 |
|  | Thyroid diagnostic burden | CPT 76536; 10005; 10021; 84443 |
| Propensity Score Matching Variables – Demographics | Age at index | Demographics |
|  | Sex | Demographics |
|  | Race (White, Black, Asian) | LOINC 2106-3; 2054-5; 2028-9 |
| Propensity Score Matching Variables – Comorbidities (ICD-10-CM) | Neoplasms | C00–D49 |
|  | Obesity | E66 |
|  | Diabetes mellitus | E08–E13 |
|  | Nicotine dependence | F17 |
|  | Ischemic heart disease | I20–I25 |
|  | Malnutrition | E40–E46 |
|  | Hypertension | I10 |
|  | Chronic kidney disease | N18 |
|  | Dyslipidemia | E78 |
|  | Cerebral infarction | I63 |
|  | Obstructive sleep apnea | G47.33 |
|  | Hyperparathyroidism | E21 |
|  | Anemia | D64 |
|  | Liver disease | K70–K77 |
|  | Hypothyroidism | E03 |
|  | Hyperthyroidism | E05 |
|  | Thyroiditis | E06 |
|  | Goiter | E04 |
|  | Iodine deficiency disorders | E01 |
|  | Osteoporosis (no fracture) | M81 |
|  | Family history of cancer | Z80 |
|  | Osteoporosis screening | Z13.820 |
| Propensity Score Matching Variables – Medication | Vitamin D supplementation | VT500 |
| Propensity Score Matching Variables – Laboratory | BMI | 9083 |
|  | Albumin | 9045 |
|  | Hemoglobin | 9014 |
|  | HbA1c | 9037 |
|  | eGFR (CKD-EPI) | LOINC 62238-1 |
|  | TSH | 9040 |
